# Synthesis of 3D nanostructured metal alloy of immiscible materials induced by megahertz-repetition femtosecond laser pulses

**DOI:** 10.1186/1556-276X-7-518

**Published:** 2012-09-21

**Authors:** Amirkianoosh Kiani, Palneet Singh Waraich, Krishnan Venkatakrishnan, Bo Tan

**Affiliations:** 1 Department of Mechanical and Industrial Engineering, Ryerson University, 350 Victoria Street, Toronto, Ontario, M5B 2K3, Canada; 2 Department of Aerospace Engineering, Ryerson University, 350 Victoria Street, Toronto, Ontario, M5B 2K3, Canada

**Keywords:** 3D nanostructured metal alloy, Femtosecond laser pulses, Aluminum and nickel oxide, Ultrafast laser thermal effects

## Abstract

In this work, we have proposed a concept for the generation of three-dimensional (3D) nanostructured metal alloys of immiscible materials induced by megahertz-frequency ultrafast laser pulses. A mixture of two microparticle materials (aluminum and nickel oxide) and nickel oxide microparticles coated onto an aluminum foil have been used in this study. After laser irradiation, three different types of nanostructure composites have been observed: aluminum embedded in nickel nuclei, agglomerated chain of aluminum and nickel nanoparticles, and finally, aluminum nanoparticles grown on nickel microparticles. In comparison with current nanofabrication methods which are used only for one-dimensional nanofabrication, this technique enables us to fabricate 3D nanostructured metal alloys of two or more nanoparticle materials with varied composite concentrations under various predetermined conditions. This technique can lead to promising solutions for the fabrication of 3D nanostructured metal alloys in applications such as fuel-cell energy generation and development of custom-designed, functionally graded biomaterials and biocomposites.

## Background

Nanoparticles generated from individual precursor materials (metals, polymers, semiconductors, dielectrics) have been shown to be useful for a lot of applications [1-8]. However, one realm of nanoparticles that has not been much studied is the capitalization of advanced properties of a group of nanoparticles in a combination to achieve a new nanostructured material/alloy with properties even superior to those of the nanoparticles of the individual materials.

Enormous attention is especially being paid to the synthesis of nanostructured metallic alloys. This is due to their unique properties suggesting their use in different applications such as energy generation applications, biomaterials for bone implants and skeletal repair, and nanostructured surfaces for cell culture and tissue growth [8-14].

Some effort has been made to synthesize one-dimensional (1D) nanostructured composite materials coated on a substrate. 1D nanofabrication methods such as ion beam mixing, sputtering, vapor deposition, thermal evaporation, laser irradiation of colloidal solutions or powder suspensions of materials, and laser ionization have been used for the synthesis of such thin-film surface deposition by composite materials [15-23]. Although these methods have some advantages, however, all these techniques are suffering from some limitations such as high fabrication cost and process time. Also, these methods can be used only for generation of nanostructured coated materials from existing alloys; to the best of our knowledge, there exists no method of producing three-dimensional (3D) nanostructured metal alloys from immiscible metals, alloys of which cannot be produced by the traditional methods.

In this research, we report, for the first time, a concept for the generation of 3D nanostructured metallic alloys by high-repetition ultrashort laser pulse irradiation of a mixture of two or more immiscible materials such as nickel oxide (NiO) and aluminum (Al) powders. In our proposed method, the 3D nanostructured metal alloy fabrication can be conducted in low processing time and cost of fabrication which can be used in applications such as renewable energy and biomedical devices. This technique does not need to have additive materials such as a liquid solution and can be conducted under ambient conditions. Also, the concentration of composite materials can be varied under our predetermined conditions; this can be achieved by varying the ratio of initial nanoparticle materials. This method can lead to a promising solution for the fabrication of nanostructured metallic alloys as a structural material or a metallic coating through laser irradiation and have far-reaching applications in the renewable energy, food service, and medical industry.

## Methods

A direct-diode-pumped Yb-doped fiber-amplified femtosecond laser with an average power of 16 W and a repetition rate ranging from 200 kHz to 26 MHz was used for this experiment. First, two powders containing microparticles of aluminum and nickel oxide, respectively, were mixed and applied onto a silicon substrate.

Second, the microparticles of aluminum were replaced by an aluminum foil and a layer of the microparticles of nickel oxide was applied onto the aluminum foil. Then, the samples were irradiated by laser pulses at a repetition rate of 8 MHz and a power of 12 W with a dwell time of 0.25 ms.

Finally, the irradiated samples were analyzed under scanning electron microscopy (SEM) and transmission electron microscopy (TEM) to determine the generation of the proposed nanostructure. An energy-dispersive X-ray spectroscopy (EDX) analysis was also carried out to probe the diffusion of the two powders to form a continuous nanostructure chain.

## Results and discussion

**Figure 1:**
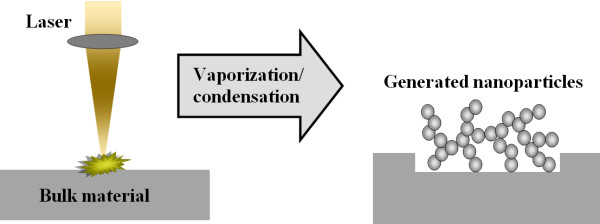
Laser ablation of a bulk material.

**Figure 2:**
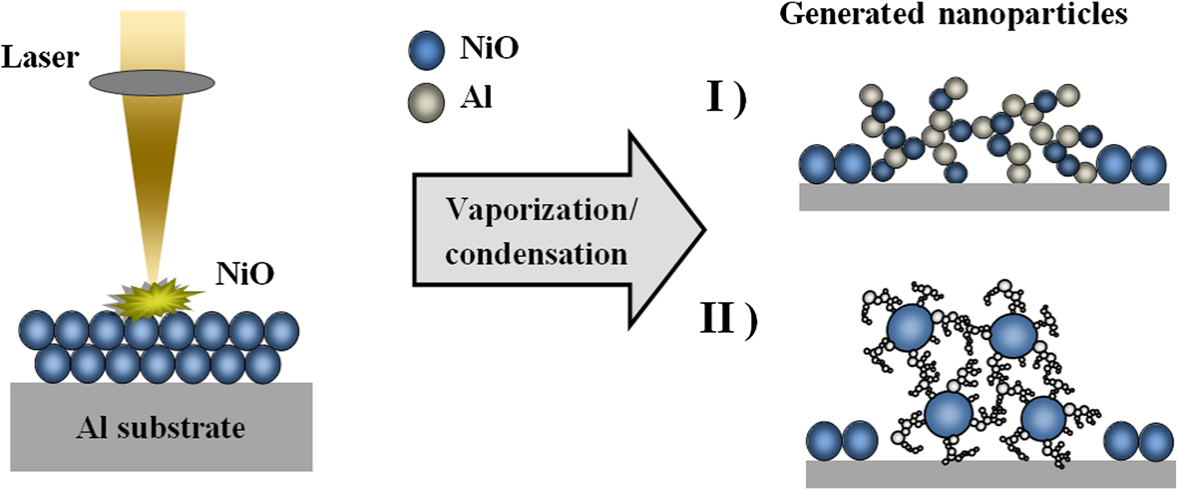
Laser ablation of a mixture of two microparticle-containing powders.

For a better understanding of the laser irradiation of a mixture of two microparticle powders, an overview of the various media that have been ablated for nanostructure generation is required. Figures 1 and 2 show an illustrative representation of laser ablation of a bulk material and a microparticle-containing powder, respectively, and the nanostructure generated in both cases.

For the case depicted in Figure 1, the laser is focused onto the top surface of the bulk material. The pulses cause ablation of the material from this top surface. Because of the densely packed nature of the target, the laser fluence for the initiation of ablation is higher, and hence, the size of the generated nanostructure is a few 100 nm in diameter.

Figure 2 shows an illustrative depiction of the laser irradiation process and the corresponding generated nanostructure composite. In this case, the laser was focused onto the powder layer; thus, a mixture of two microparticle-containing powders was ablated and the nanostructure generated was analyzed. Due to the absence of the dense packing, the laser fluence for the ablation of the powder layer was lower in comparison to that with a solid target: a reduction of 3.5 times in laser fluence was observed. The observed nanostructure was finer in comparison to that for the solid target: the average size was in the range of 60 to 90 nm.

**Figure 3:**
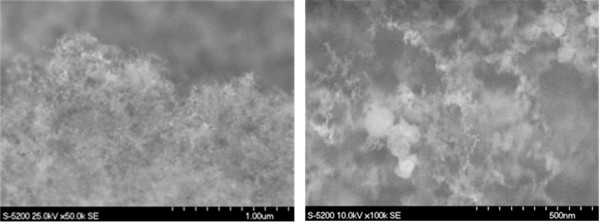
SEM images of generated microstructure.

The formation of the 3D structure can be attributed to the lack of photo-ionization in the generated nanoparticles, thus facilitating the agglomeration of the nanoparticles into a 3D nanostructure. The shock wave that travels through the sample, as a result of irradiation with laser pulses, causes rapid condensation of the particles, thereby forming even finer nanoparticles and eventually very fine nanostructures. From Figure 3, the size of the agglomerated nanoparticles can be determined to be of the order of a few nanometers.

**Figure 4:**
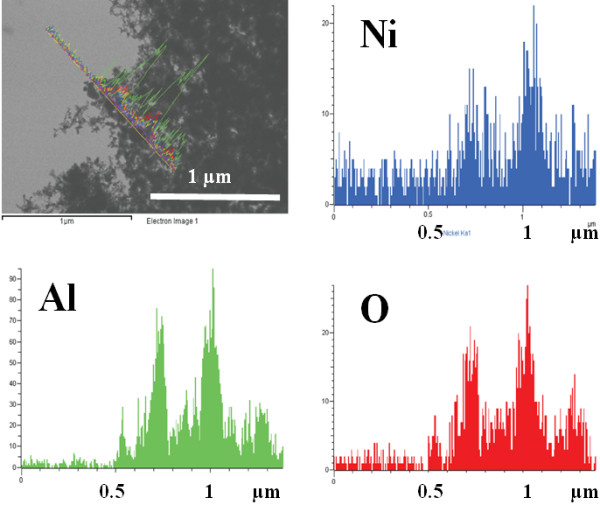
EDX analysis result confirming presence of Al and Ni in the nanostructure.

Figure 4 presents the result of the EDX analysis carried on the sample. The EDX analysis confirmed the presence of both aluminum and nickel in the obtained nanostructured metal alloy.

**Figure 5:**
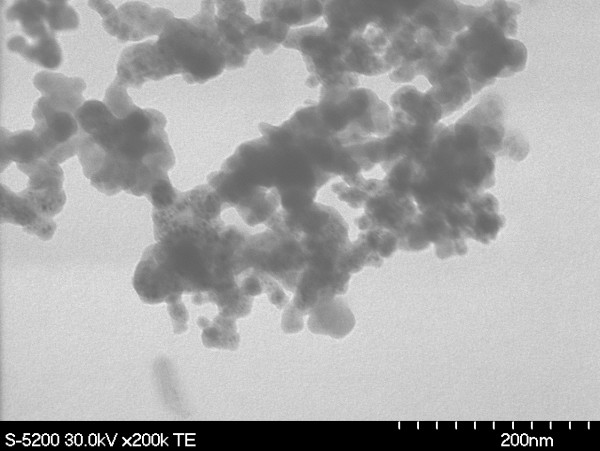
TEM image of the generated nanoparticle network.

A TEM analysis was also carried out on the ablated samples to determine the extent and type of mixing of the two powders through laser irradiation. Figure 5 shows the TEM image of the ablated microparticle mixture.

**Figure 6:**
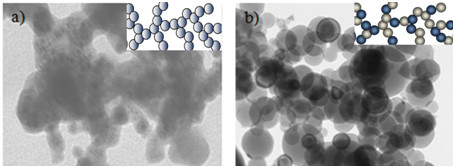
**TEM images of generated nanostructures. **(**a**) Center of ablation area: aluminum nanoparticles embedded in a background of nickel. (**b**) Away from the center of the ablation: interconnected chains of aluminum and nickel oxide.

Figure 6 shows a closer comparison between the mixing at the center and away from the center of the focused laser beam (ablation center): 

1. Nanoparticles fused to form 3D nanostructures (Figure 6a) at the center of the ablated area.

2. Aluminum particles embedded in nickel nuclei (Figure 6b) away from the center of the ablated area.

The difference in the mixing is clearly visible in the case of Figure 6a in which the aluminum nanoparticles are embedded in a background of nickel, while in Figure 6b, aluminum and nickel oxide form interconnected chains and do not show signs of mixing between the two in the plasma state.

In order to explain the post-ablation mixing of the two powders, the fundamental theory behind the process of laser ablation for material removal has to be reexamined. The method of material removal by laser ablation has been explained by heating of the target material above its boiling temperature induced by laser pulses, followed by rapid cooling once the laser pulses stop. When ablation of the target material is carried out in a background gas environment or in ambient air, the presence of the air/gas causes the redeposition of the ablated material onto the target surface, which does not take place for laser ablation in a vacuum [24].

**Figure 7:**
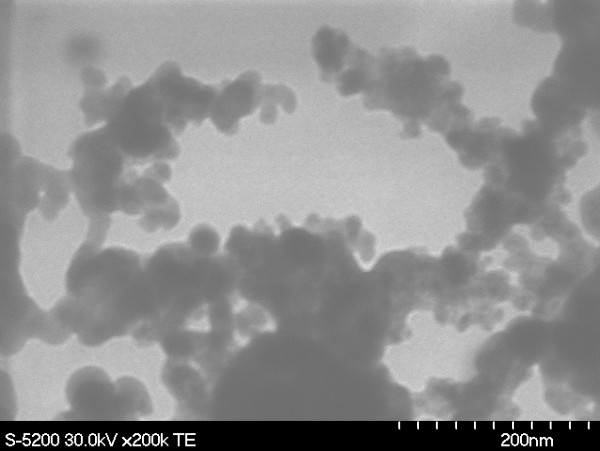
TEM image showing Al embedded in Ni nuclei.

Due to the presence of two powders of two different chemical compositions, there is a difference in the boiling point of the two. This difference in the boiling point is a factor during the cooling phase that follows after the laser pulses have stopped hitting the target. As per the material data for aluminum and nickel oxide, there is a huge gap in the boiling point temperature for the two materials. Nickel has a higher boiling point temperature (2,730°C) than aluminum (2,327°C). Thus, after the laser pulses stop, during the rapid cooling phase, the nickel oxide solidifies faster than aluminum. The cooled nickel oxide provides nucleation sites for the cooling aluminum. This results in the deposition of the aluminum nanoparticles on nickel oxide nuclei, clearly observed in the TEM image shown in Figure 7.

**Figure 8:**
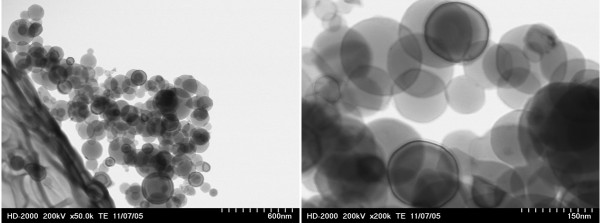
TEM images of the generated nanostructure.

Another aspect of laser ablation that has been recently highlighted is the presence of a temperature gradient that exists across the surface of the target material [25]. The temperature at the point where the laser directly hits is the highest, and it decreases as we move away from the center. There is also the existence of isotherms across the target surface [25]. Taking into account the above temperature gradient, the layer of the mixture of aluminum and nickel oxide powders will be subjected to different temperatures depending on the position from the point of laser impact. Thus, there exists a variation in the extent of mixing of the two powders after laser ablation. Figure 8 shows the TEM images of the nanostructure generated in the area away from direct impact of the laser.

**Figure 9:**
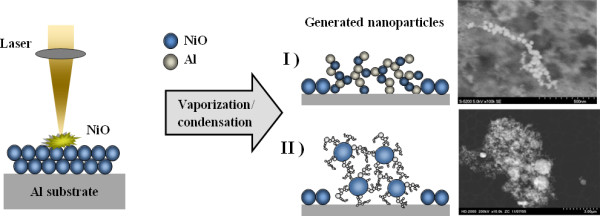
Laser ablation of NiO microparticles applied on Al substrate.

In extension of the above research, the microparticles of aluminum were replaced by an aluminum foil. A layer of the microparticles of nickel oxide was applied onto the aluminum foil. The sample was then ablated and analyzed for nanostructure generation. The process is illustrated in Figure 9.

**Figure 10:**
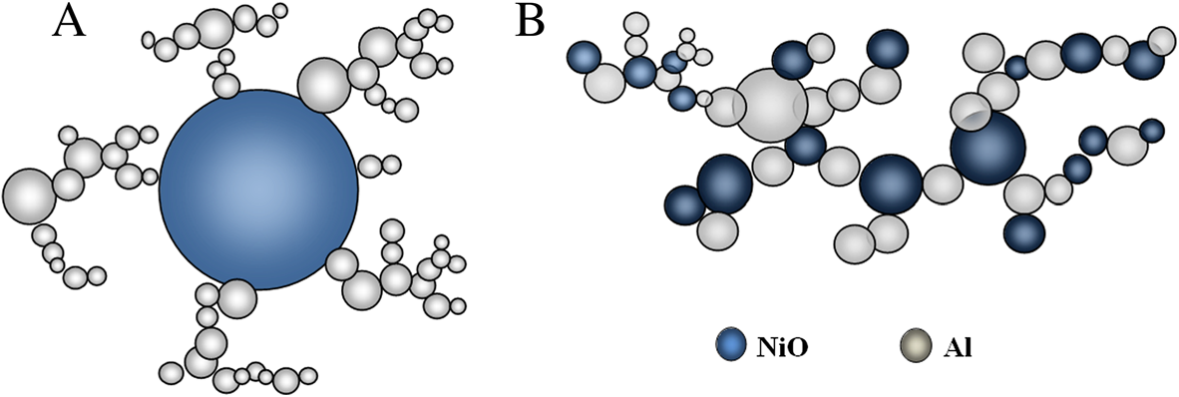
**Two types of generated nanostructured metal alloys. **(**A**) Aluminum nanoparticles generated on micro-NiO particles (away from the center of the ablated area). (**B**) Nanoparticles fused to form 3D nanostructures (at the center of the ablated area).

**Figure 11:**
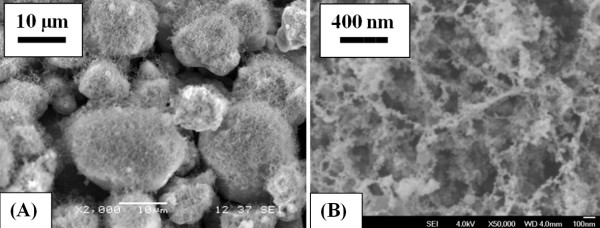
**SEM images of nanostructures generated by laser ablation of NiO microparticles on Al foil. **(**A**) Away from the ablated area. (**B**) At the center of the ablated area).

For the current scenario, as shown in Figure 9, the laser was focused so as to ablate the microparticle layer and the aluminum foil simultaneously. The particles from the foil and the microparticle layer were ejected into the plume and, upon subsiding of the laser pulses, formed into nanoparticle networks. The generated networks showed two types of generated nanostructured metal alloys as follows: 

1. Aluminum nanoparticles grown on micro-NiO particle (Figures 10A and 11A) away from the center of the ablated area.

2. Nanoparticles fused to form 3D nanostructures (Figures 10A and 11B) at the center of the ablated area.

Figure 11 shows the SEM images of nanostructure materials obtained by the ablation of NiO microparticles coated on an aluminum foil in the ablated area (Figure 11B) and away from the ablated area (Figure 11A). As shown in Figure 11A, microparticles of NiO (away from the ablated area) were covered by aluminum nanoparticles; however, in the ablated area, the particles from the foil and the microparticle layer were ejected into the plume and, upon subsiding of the laser pulses, formed into nanoparticle networks. The generated networks showed a certain extent of mixing between the two materials.

**Figure 12:**
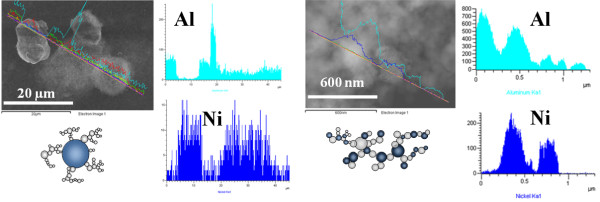
EDX result of generated nanostructured induced by laser pulses.

In an effort to verify the elemental composition of the fabricated micro/nanostructures, an EDX analysis was conducted. In Figure 12 (left), the EDX results clearly show the presence of nickel in microparticles as well as a trace of Al in nanostructured materials which are believed to be made of Al. Additionally, EDX results of the ablated area show the existence of fused Al-NiO nanoparticles at the center of the ablated area (Figure 12, right).

## Conclusions

In the current study, the process of laser ablation of microparticles for generating a 3D nanostructured metallic alloy has been successfully applied for the generation of 3D nanostructure materials through laser irradiation of a mixture of aluminum and nickel oxide microparticle-containing powders and microparticles of nickel oxide applied on the aluminum substrate. Apart from the generation of the nanostructure composites, mixing between the two powders is also observed. Three different types of mixing are observed: one where aluminum is embedded in a pool of nickel oxide, another where nickel and aluminum are present in an agglomerated chain of their nanoparticles, and finally, aluminum nanoparticles generated on micro-NiO particles (away from the center of the ablated area). The mixing has been explained by the difference in the boiling point of the two powders and its effect during the rapid cooling phase after the end of the laser pulse train. Also, the formation of these structures and the concentration of composite materials can be varied under our predetermined conditions; this can be achieved by varying the ratio of initial nanoparticle materials.

This mixing process opens up new possibilities where powders of different materials can be ablated to produce a mixture, the materials combining at the nanoscale. This can also be applied for 3D nanostructured metallic alloy formation where two or more immiscible materials can be combined at the nanoscale to form an alloy. Another area where this technique can be used is the combination of a bulk material with a material in the powder phase.

The process is single step and very flexible as it can produce nanoparticles or nanoporous materials of different compositions such as nanoscale metals and metal oxides which can be used in energy generation applications. Also, this new technique can lead to promising solutions for development of better biomaterials and biocomposites for custom-designed, functionally graded bone implants and skeletal repair and also for the nanostructured surfaces for cell culture and tissue growth.

## Competing interests

The authors declare that they have no competing interests.

## Author’s contributions

AK and PSW carried out the laser processing of the samples and the characterization and drafted the manuscript. KV and BT conceived of the study and participated in its design and coordination. All authors read and approved the final manuscript.
